# Stress, Eating Behavior and Adverse Health in Parents of Young Children with Autism Spectrum Disorder

**DOI:** 10.1007/s10803-022-05825-3

**Published:** 2022-11-25

**Authors:** Anna van der Lubbe, Hanna Swaab, Robert R. J. M. Vermeiren, Wietske A. Ester

**Affiliations:** 1 Sarr Expert Centre for Autism, Youz Child- and Adolescent Psychiatry, Parnassia Group, Dynamostraat 18, Rotterdam, The Netherlands; 2 Clinical Neurodevelopmental Sciences, Leiden University, Wassenaarseweg 52, Leiden, The Netherlands; 3 Parnassia Group, Parnassia Academy, Den Haag, The Netherlands; 4 Curium-LUMC, Child- and Adolescent Psychiatry, Endegeesterstraatweg 27, Oegstgeest, The Netherlands; 5 Leiden Institute for Brain and Cognition, Leiden University, Leiden, The Netherlands

**Keywords:** Autism Spectrum Disorder, Parenting Stress, Eating Behavior, Adverse Health, Early Childhood, Fathers

## Abstract

Mothers of children with Autism Spectrum Disorder (ASD) often experience chronic stress and are at risk for adverse health. However, little is known about fathers, especially when their child is in early childhood. Parenting stress, eating behavior and physical health was evaluated in mothers (n = 48) and fathers (n = 43) of young children (3–7 years) with ASD by questionnaires and physical measurements. Mother’s prevalence rates of obesity (39.1%), abdominal obesity (59.6%) and metabolic syndrome (21.6%) were higher than the norm. In fathers, the prevalence rate of clinical parenting stress (33%) was higher than the norm. Parenting stress was positively related to disinhibited eating in mothers, not in fathers. It is crucial to monitor stress and health of parents of children with ASD.

Parents of children with autism spectrum disorder (ASD) report considerable higher levels of stress than parents of neurotypical children and parents of children with other neurodevelopmental conditions, like Cerebral Palsy or Down Syndrome (Davis & Carter, 2008; Hayes & Watson, 2013; Hoffman et al., 2009). Stress is a biological and psychological adaption to demanding circumstances. Although a certain amount of stress is common in all caregivers, parenting stress may shift towards chronic stress if parenting stress is persistent and overwhelming. ASD is characterized by difficulties in social communication and social interaction and restricted and repetitive patterns in behaviors, interest and activities (American Psychiatric Association, 2013). Especially these core symptoms in ASD may be particularly stressful for parents. For example, studies have found high associations between experienced stress levels in parents of children with ASD and impairments in social relatedness and repetitive behaviors in the child (Davis & Carter, 2008; Gabriels et al., 2005).

Chronic stress is associated with multiple serious conditions, including obesity, metabolic syndrome and cardiovascular disease (Candola et al., 2006; Low, Salomon, & Matthews, 2009; Tomiyama, 2019). Moreover, chronic stress is related to overeating and unhealthy eating (Adam & Epel, 2007; Torres & Nowson, 2007). As parents of children with ASD experience considerable stress, they may be at risk for adverse health. Previous studies demonstrated that mothers of children with ASD reported a worse physical condition than parents of neurotypical children or children with other developmental disabilities, such as Down Syndrome (Allik et al., 2006; Fairthorne et al., 2015; Smith et al., 2013). Further insight into the connection between stress, eating behavior and adverse health may not only have implications for parents of a child with ASD, but also for parents with stress related unhealthy eating behavior in general.

Surprisingly, research into the relation between stress, eating behavior and adverse health in parents of children with ASD is sparse. A previous study showed that mothers of children with ASD report higher levels of perceived stress, greater reward-based eating and have a worse metabolic health compared to mothers of typically developing children (Radin, 2019). It was hypothesized that on the short term, chronic stress related to caring for a child with ASD promotes greater consumption of highly palatable foods in order to temporarily decrease negative affect, which may promote weight gain and subsequent worsening of metabolic components, such as LDL cholesterol, on the long-term. However, the impact of stress on eating behavior and adverse health of parents of children with ASD in early childhood is unknown, especially in fathers.

Traditionally, research on the effects of ASD in parents is directed at mothers, while fathers of these children are rarely included. Studying stress in both parents of children with ASD is important as raising a child with ASD might affect mothers and fathers differently, due to psychological, biological and social factors. First, there are differences between mothers and fathers in how they perceive their child’s problem behaviour. For example, a study in parents of toddlers with ASD suggested the degree to which behaviour problems and competencies are perceived as stressful varies between mothers and fathers (Davis & Carter, 2008). In addition, mothers of a child with ASD report more positive experiences compared to fathers of a child with ASD (Kayfitz et al., 2010). Second, there are biological differences between mothers and fathers. For example, a study in healthy men and women demonstrated higher hair cortisol levels in males compared to females, which may indicate a different physiological expression of long-term stress (Dettenborn et al., 2012). Lastly, raising a child with ASD might affect mothers and fathers differently due to social differences. For example, mothers of children with ASD spend 26% more time in childcare than fathers of a child with ASD, while fathers spend 41% more time in paid employment than mothers(Hartley et al. 2014). The few studies that have been performed in fathers, indicate a high level of parenting stress in fathers too: for example, Davis and Carter (2008) reported 39% of the mothers and 28% of the fathers of a child with ASD scoring above the 90th percentile for parenting stress. As mothers and fathers both play an important role in their child’s life, it is important to include both parents in studies of impact of parenting stress.

As every developmental stage of a child comes with different challenges, stress experienced by parents may differ across the years. To illustrate, some studies suggest that the level of parenting stress decreases as the child becomes older (Neece et al., 2012). Some challenges that are specific to the early childhood, may be particularly stressful for parents. For example, at this developmental stage, children have to master different developmental challenges like motor milestones, language development and learning to socially interact and meet the challenge of participation in primary education. In addition, in the early school years children are often diagnosed with ASD and parents are searching for the appropriate approach for their child, since the mean age of children under the age of 10 receiving their ASD diagnosis is 43.2 months (Van ‘t Hof et al., 2021). Also, parents often have more than one child in their young family needing physical and emotional care during this period, while at the same time they must meet expectations in their careers as well (Frenken, 2015).

Especially during the early developmental stage of a child, it might be important to evaluate the early associations between stress, eating behavior and adverse health in parents, since these early associations may lay foundation for the health of parents later in life. To illustrate, a longitudinal study in middle-aged men showed a relationship between components of metabolic syndrome, such as obesity, hypertension and elevated cholesterol with all-cause mortality and cardiovascular mortality 13.6 years later (Ho et al., 2008). As components of metabolic syndrome, such as (abdominal) obesity, hypertension and dyslipidemia are associated with chronic stress, these components may function as an appropriate measure for stress-related health problems in parents of a child with ASD (Bergmann et al., 2014; Dijkstra- de Neijs et al., 2020). By focussing on parenting stress, eating behaviour and adverse health components in parents of young children with ASD, it is possible to investigate early processes that may have lasting impact. In the current study a homogenous group of parents of young children between 3 and 7 years will be included to explore the impact of raising a young child with ASD, to investigate early associations between stress, eating behaviour and adverse health.

The current study will focus on stress, eating behavior and adverse health in both mothers and fathers of young children with ASD. The first goal of the current study is to investigate whether mothers and fathers of a young child with ASD display problematic levels of stress, eating behavior and adverse health. The second goal of this study is to explore whether stress is related to eating behavior and adverse health in both mothers and fathers of a child with ASD.

## Methods

### Procedure

The current study is a cross-sectional study investigating stress, eating behavior and adverse health in parents of young children with ASD. This study is part of the ongoing Tandem Study (Dutch Trial register: NL7534), a longitudinal study on the developmental impact of ASD and the effectivity of treatment, approved by the Institutional Review Board of the Leiden University Medical Center, The Netherlands.

### Participants

**Fig. 1:**
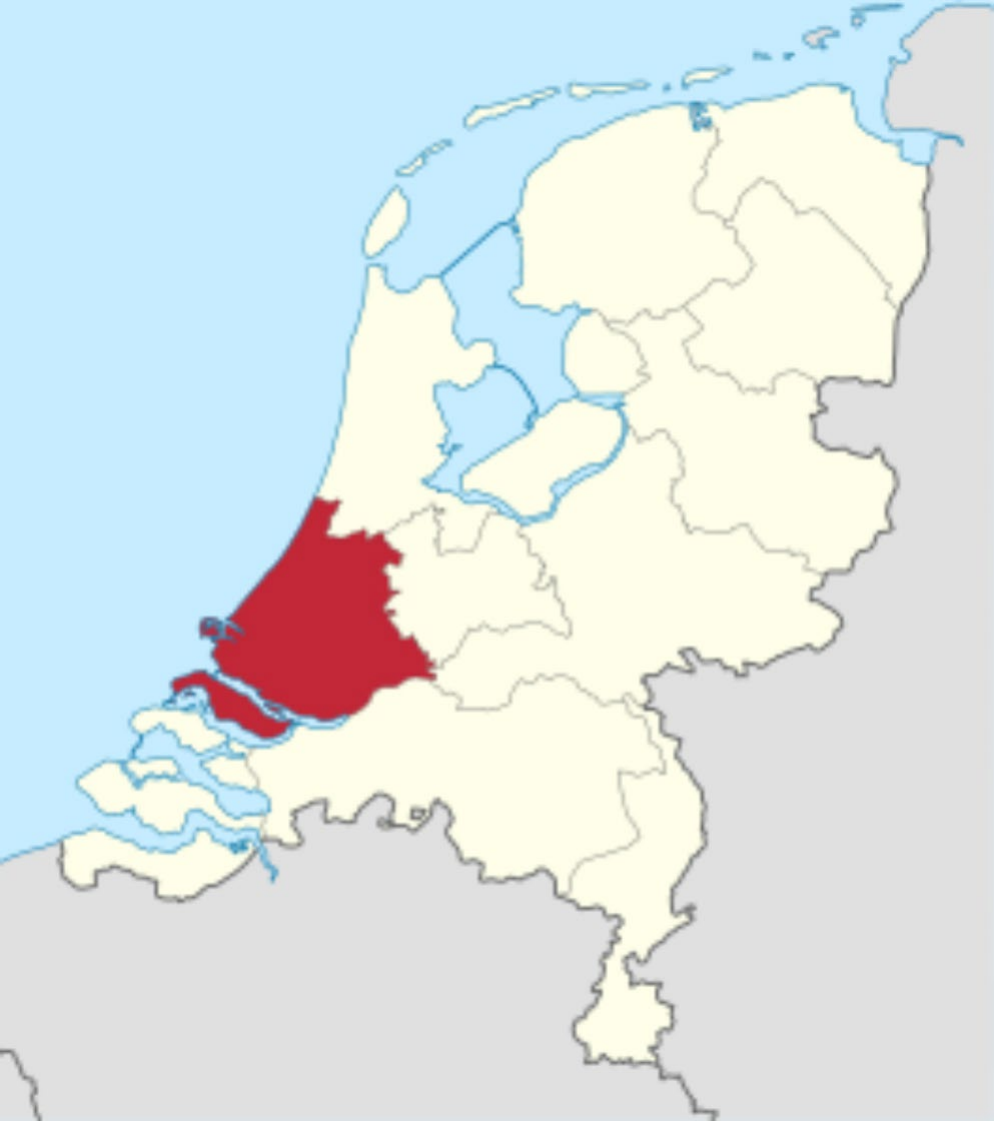
*Geographical map of the study’s location*

Parents were recruited from Youz Parnassia Group and GGZ Delfland, both mental health care providers in The Netherlands. Parents were eligible for inclusion if: (1) their child was diagnosed with ASD and (2) their child was aged between 3 and 7 years. If parents were eligible for inclusion and agreed to be contacted by the research team, parents received an oral and written description of the study. If parents decided to participate in the study, they met with a researcher to complete the informed consent process. Data-collection took place at the study facility (Sarr Expertise Center for Autism) located in Rotterdam, the Netherlands, or at participant’s homes, all located in South-Holland (see Fig. 1 for a geographical map). In total, 91 parents (48 mothers and 43 fathers) of 50 children participated in this study.

### Measures

#### Stress

Parenting stress was measured using the Parenting Stress Questionnaire (In Dutch: *Opvoedbelasting Vragenlijst* [OBVL]). The OBVL is a 34-item self-report measure of parenting stress (Vermulst et al., 2015). Items are answered on a 4-point Likert scale from 1 (“Not true”) to 4 (“Very true”). For this study, the total score on the OBVL was used (*α* **=** 0.91), in which a high score reflects a high level of parenting stress. As the response rate of fathers in the standardization study of the OBVL was minimum, the norm data of the OBVL is based on the response of mothers. For mothers and fathers in our sample, t-scores were derived from the norm tables based on data of 848 Dutch mothers of neurotypical children aged between 0 and 11 years (Vermulst, 2015). Based on their t-scores, parents were classified in one of the three following categories (1) Normal (t-score < 60), (2) Sub-clinical (t-score = 60–63) and (3) Clinical (t-score ≥ 64).

#### Eating Behavior

Eating behavior was measured using the Dutch Eating Behaviour Questionnaire (DEBQ). The DEBQ is a 33-item self-report measure of eating behavior consisting of 3 subscales: Emotional eating, External eating and Restrained eating (van Strien, 2015). The subscale ‘Emotional eating’ refers to eating in response to emotions (e.g., ‘do you have the desire to eat when you are anxious, worried or tense?’). The subscale ‘External eating’ refers to eating in response to external food cues (e.g., ‘If you walk past a snackbar, do you have the desire to buy something delicious?’). The subscale ‘Restrained eating’ refers to limiting food-intake to lose weight (e.g., ‘Do you deliberately eat less in order not to become heavier?’). Items are scored on 5-point Likert scale from 1 (“never”) to 5 (“very often”). Higher subscale scores indicate a higher level of emotional, external or restrained eating. Cronbach’s alpha value is 0.96 for Emotional eating, > 0.78 for External eating and > 0.90 for Restrained eating. Based on existing norms, participants were classified into two categories: (1) Normal and (2) High (≥ 80th percentile). Age and gender-appropriate norms were used of males (n = 815) and females (n = 921) aged between 21 and 70 years from the Dutch general population (van Strien, 2015).

#### Body Mass Index, Waist Circumference and Blood Pressure

Body height was measured by a stadiometer (Seca 213) and weight by a digital scale (Seca Clara 803). Body Mass Index (BMI) was calculated by dividing weight in kilograms by the square of height in meters. Participants were classified into three BMI classes: normal weight (< 25 kg/m^2^), overweight (25–30 kg/m^2^) and obesity (≥ 30 kg/m^2^). The percentage of participants in each category was compared to Dutch males aged 18–59 (n = 27,991) and Dutch females aged 18–49 (n = 29,650) from the population-based Lifelines Cohort Study (Slagter et al., 2017).

Waist circumference (cm) was measured at the lowest point of the lowest rib and the upper border of the pelvic crest using a measuring tape. Waist circumference was also used to measure abdominal obesity. Abdominal obesity was defined according to the recommendations of the World Health Organization (WHO), with a waist circumference of 102 cm or higher for males and 88 cm or higher for females (WHO, 2008). The percentage of participants in each category was compared to Dutch males aged 18–59 (n = 27,991) and Dutch females aged 18–49 (n = 29,650) from the population-based Lifelines Cohort Study (Slagter et al., 2017).

We measured systolic and diastolic blood pressure using a blood pressure monitor (Omron M6). During the blood pressure measurement, parents were asked to sit still and to not speak. Blood pressure measures were done twice, and the average of the two measures was calculated. We used systolic and diastolic blood pressure to define an elevated blood pressure as follows: systolic blood pressure ≥ 130 and/or diastolic blood pressure  ≥  85 and/or use of antihypertensive drugs. Prevalence rates of elevated blood pressure in our sample were compared with prevalence rates from Dutch males aged 18–59 (n = 27,991) and Dutch females aged 18–49 (n = 29,650) from the LifeLines cohort study (Slagter et al., 2017).

#### Cholesterol, Triglycerides and Glucose

To measure cholesterol, triglycerides and glucose values, blood samples (18 ml) were drawn from participants by a phlebotomist. Parents were instructed to not eat or drink anything other than water for 8 h before the blood test. After collection, the samples were sent to the IIselland Hospital for laboratory analysis.

#### Metabolic Syndrome

According to the R-NCEP-ATPIII (R-APTIII), at least three of the five metabolic risk components need to be present to diagnose MetS (Grundy et al., 2005). These metabolic risk components include: (1) elevated waist circumference (≥ 102 cm in men, ≥ 88 in women), (2) elevated triglycerides (≥ 1.7 mmol/L) and/or use of triglyceride-lowering medication-lowering medication (3) reduced HDL Cholesterol (< 1.03 mmol/L in men, < 1.3 mmol/L in women) and/or use of lipid-lowering medication, (4) elevated blood pressure (systolic blood pressure ≥ 130 and/or diastolic blood pressure ≥ 85 and/or use of antihypertensive drugs) (5) elevated fasting glucose (≥ 5.6 mmol/L) and/or use of glucose-lowering medication. We compared prevalence rates of metabolic syndrome in our sample with the prevalence rates of metabolic syndrome in same-aged males and females from the Dutch Lifelines cohort study (Slagter et al., 2017).

#### Smoking Behavior

To estimate smoking behavior, parents filled in a demographic questionnaire. Participants were asked whether they smoked. Three answer options were possible: (1) Yes (2) No, I never smoked and (3) No, I stopped. If parents indicated to smoke, they were asked how many cigarettes they smoke during a day. If parents stopped smoking, they were asked for how long they smoked and how many cigarettes per day they smoked. Smoking status was compared to the percentage of smokers in males and females aged 18–79 from the population-based Lifelines Cohort study (Slagter et al., 2017).

#### Demographic Variables

Parental educational levels were obtained by questionnaire and categorized as follows: low (primary education, lower vocational secondary education or lower secondary education), middle (intermediate vocational education, intermediate secondary education and higher Secondary education) and high (higher vocational education and university). Parents also filled in their marital status (married/cohabiting versus single parent), who was the primary caregiver and whether they had paid employment.

#### Measurements Before or During COVID-19

As measurements in part of our sample were taken during the COVID-19 outbreak, we included an extra variable to control for this. In the Netherlands, the government took measures, including closing of all primary schools, on March 15th 2020 to prevent the further spread of COVID-19. All parents who filled in the questionnaires before March 15th 2020 were considered ‘Before COVID-19’ and all parents who filled in the questionnaires from March 15th 2020 were considered ‘During COVID-19’.

### Statistical Analyses

The variables ‘OBVL total’, ‘NVE emotional eating’, ‘NVE restrained eating’, ‘cholesterol HDL’ and ‘Triglycerides’ were not normally distributed. Therefore, non-parametric tests were performed to evaluate these variables.

To control for parenting stress due to COVID-19, we tested whether there were differences in parenting stress between parents who participated before COVID-19 and during COVID-19 using a Mann-Whitney U test.

If height or weight data were missing, the missing value was replaced by the self-reported height and weight of the participant. Self-reported height and self-reported weight strongly correlated with measured height (*r* = .98, *p* < .001) and weight (*r* = .97, *p* < .001) in participants of which we had both self-reported and measured height (n = 68) and weight (n = 64). For the other variables, missing values were treated using pairwise deletion.

To investigate whether mothers and fathers of a young child with ASD display problematic levels of stress, eating behavior and adverse health, we used Chi-Square Goodness of Fit tests were performed to determine whether the proportion of parents scoring above a certain cut-off was different from the general population. For parenting stress, we also performed a Mann-Whitney U test to test whether there were differences in parenting stress (OBVL) between mothers and fathers of a young child with ASD regarding parenting stress.

To investigate whether stress is related to eating behavior and adverse health, we performed a Pearson’s correlation analysis. For the variables that were not normally distributed, we performed a Spearman’s correlation analysis. All analyses were performed in SPSS Statistics 25.

## Results

### Descriptives

In total, 91 parents (48 mothers and 43 fathers) of 49 young children with ASD (3–7 years) participated in the study. Parents were aged between 23 and 58 years old (*Mean* = 34.9, sd = 6.0). The mother was the primary caregiver in 48 families (98%) and the father was the primary caregiver in 1 family (2%). Parents were married or co-habiting in 36 families (73.5%), the parent was a single parent in 8 (16.3%) of the families and marital status was missing in 5 families (10.2%).

Three (6.3%) mothers had a low educational level, 21 (43.8%) had a middle educational level and 14 (29.2%) had a high educational level, and highest completed education was missing in 10 mothers (20.8%). In fathers, highest completed education was low in 9 (20.9%), middle in 17 (39.5%) and high in 9 (20.9%) and educational level was missing in 8 (18.6%). In total, 75% of the mothers and 97.7% of the fathers had paid employment.

In total, 36 (73.5%) families participated before COVID-19 and 13 families (16.3%) participated after COVID-19. There was no difference in parenting stress between parents who participated before COVID-19 and after COVID-19, (U = 520,5, *z* = − 0.68, *p* = .50).To our knowledge, one mother in our sample was pregnant during this study. However, excluding this mother from analysis did not make a difference in results regarding obesity and waist circumference. Therefore, analyses were performed including this mother.

### Stress in Mothers and Fathers of a Child with ASD

#### Stress

As shown in Table 1, more than half of the mothers of a child with ASD scored above the 90th percentile for parenting stress, which is significantly more than mothers of neurotypical children (*χ*^*2*^(2) = 83.88, *p* < .01). In addition, 33% of the fathers in our sample scored above the 90th percentile for parenting stress, which is threefold the number of the reference mothers from the general population (*χ*^*2*^(2) = 24.42, *p* < .01). Within our sample, mothers (Median = 63.5, IQR = 21) reported significantly more parenting stress (U = 522,5, *z* = -2.34, *p* = .02) than fathers (Median = 55.5, IQR = 15.13).

**Table 1:** *Stress, eating behavior and adverse health in parents of a child with ASD compared to males and females from the general population*

		Parents of child with ASD			ASD parents vs. norm group			
			**N**	**%**	**Expected %**	**Chi-square**	***P***	**Comparison group**
**Stress**								
Parenting stress (OBVL)								
*Mothers*						83.88	< 0.001	Dutch mothers (n = 848) of neurotypical children aged between 0–11 (Vermulst, 2015).
	Normal (t-score < 60)		13	31	85			
	Sub-clinical (t-score 60–63)		7	16.7	5			
	Clinical (t-score ≥ 64)		22	52.4	10			
*Fathers*						24.42	< 0.001	Dutch mothers (n = 848) of neurotypical children aged between 0–11 (Vermulst, 2015).
	Normal (t-score < 60)		17	47.2	85			
	Sub-clinical (t-score: 60–63)		7	19.4	5			
	Clinical (t-score ≥ 64)		12	33.3	10			
**Eating behaviour**								
Emotional eating (NVE)								
*Mothers*						0.95	N.S.	Females (n = 1143) from the Dutch general population aged between 21 and 70 years (van Strien, 2015)
	Normal		31	73.8	80			
	High (≥ 80th percentile)		11	26.2	20			
*Fathers*						2.97	N.S.	Males (n = 807) from the Dutch general population aged between 21 and 70 years (van Strien, 2015)
	Normal		33	91.7	80			
	High (≥ 80th percentile)		3	8.3	20			
External eating (NVE)								Females (n = 1143) from the Dutch general population aged between 21 and 70 years (van Strien, 2015) Males (n = 807) from the Dutch general population aged between 21 and 70 years (van Strien, 2015)
*Mothers*						1.82	N.S.	
	Normal		30	71.4	80			
	High (≥ 80th percentile)		12	28.6	20			
*Fathers*						0.55	N.S.	
	Normal		27	75	80			
	High (≥ 80th percentile)		9	25	20			
Restrained eating (NVE)								Females (n = 1143) from the Dutch general population aged between 21 and 70 years (van Strien, 2015)
*Mothers*						0.29	N.S.	
	Normal		35	83.3	80			
	High (≥ 80th percentile)		7	16.7	20			
*Fathers*						0.11	N.S.	Males (n = 807) from the Dutch general population aged between 21 and 70 years (van Strien, 2015)
	Normal		28	77.8	80			
	High (≥ 80th percentile)		8	22.2	20			
**Adverse health**								
Body Mass Index								
*Mothers*						23.03	< 0.001	Dutch females (n = 29,650) aged 18–49 from the population-based LifeLines cohort study (Slagter et al., 2017)
	Normal weight		18	39.1	55.9			
	Overweight		10	21.7	29.8			
	Obesity		18	39.1	14.3			
*Fathers*						3.61	N.S.	Dutch males (n = 27,991) aged 18–59 from the population-based LifeLines cohort study (Slagter et al., 2017).
	Normal weight		11	26.2	40.6			
	Overweight		24	57.1	46.5			
	Obesity		7	16.7	12.9			
Waist circumference								Dutch females (n = 29,650) aged 18–49 from the population-based LifeLines cohort study (Slagter et al., 2017)
*Mothers*						10.25	< 0.01	
	Abdominal obesity		28	59.6	37			
*Fathers*						0.59	N.S.	Dutch males (n = 27,991) aged 18–59 from the population-based LifeLines cohort study (Slagter et al., 2017).
	Abdominal obesity		11	26.8	21.9			
Blood pressure								Dutch females (n = 29,650) aged 18–49 from the population-based LifeLines cohort study (Slagter et al., 2017)
*Mothers*						0.37	N.S.	
	Elevated blood pressure		12	25.5	21.8			
*Fathers*						2.06	N.S.	Dutch males (n = 27,991) aged 18–59 from the population-based LifeLines cohort study (Slagter et al., 2017).
	Elevated blood pressure		25	61	49.7			
Metabolic syndrome								Dutch females (N = 29,650) aged 18–49 from the population-based LifeLines cohort study (Slagter et al., 2017).
*Mothers*						8.40	< 0.01	
	Yes		8	21.6	8.4			
*Fathers*						0.53	N.S.	Dutch males (N = 27,991) aged 18–59 from the population-based LifeLines cohort study (Slagter et al., 2017).
	Yes		7	22.6	17.6			
Smoker								Dutch females (n = 41,075) aged 18–79 from the population-based LifeLines cohort study (Slagter et al., 2017).
*Mothers*						0.46	N.S.	
	Yes		11	23.9	19.9			
*Fathers*						0.88	N.S.	Dutch males (n = 32,189) aged 18–79 from the population-based LifeLines cohort study (Slagter et al., 2017).
	Yes		12	30	23.7			

#### Eating Behavior

As shown in Table 1, there was no significant difference in prevalence rates of emotional eating, external eating and restrained eating between parents of a child with ASD and same-aged individuals in the Dutch Lifelines cohort study.

#### Adverse Health

As displayed in Table 1, more than 39% of the mothers in our sample were obese.

This percentage is almost three times the percentage of obesity in same-aged females from the Dutch Lifelines cohort study (*χ*^*2*^(2) = 23.03, *p* < .01). In addition, almost 60% of the mothers of a child with ASD had abdominal obesity, which is about 1.5 times more often than females from the Lifelines cohort study (*χ*^*2*^(1) = 10.25, *p* < .01). Furthermore, approximately 22% of the mothers in our sample fulfilled criteria for metabolic syndrome (at least three of the five metabolic risk components), which was about 2.5 times higher than in same-aged females from the Dutch Lifelines cohort study (*χ*^*2*^(1) = 8.40, *p* = < 0.01). There was no significant difference in prevalence of an elevated blood pressure (*χ*^*2*^(1) = 0.37, *p* = .54) or smoking status (*χ*^*2*^(1) = 0.46, *p* = .50) between mothers in our sample and the females from the Lifelines Cohort study.

In fathers, there were no significant differences between fathers in our sample and same-aged males from the Lifelines cohort study regarding obesity (*χ*^*2*^(2) = 3.61, *p* = .17), abdominal obesity (*χ*^*2*^(1) = 0.59, *p* = .11) blood pressure (*χ*^*2*^(1) = 2.06, *p* = .15), metabolic syndrome (*χ*^*2*^(1) = 0.53, *p* = .47) and smoking status (*χ*^*2*^(1) = 0.88, *p* = .35).

### Correlations Between Stress, Eating Behavior and Adverse Health

As shown in Table 2, mothers who reported a higher level of parenting stress, reported a significantly higher level of emotional eating (*r* = .53, *p* < .01) and external eating (*r* = .47, *p* < .01). This association was not found in fathers. There was no significant relationship between parenting stress and adverse health outcomes in mothers or fathers.

**Table 2:** *Correlations between stress, eating behavior and metabolic health in mothers and fathers of children with ASD within the Tandem study (n = 91)*

	Mothers Parenting stress (OBVL)	Fathers Parenting stress (OBVL)
Emotional eating (NVE) ^a^	0.53**	0.05
External eating (NVE)	0.47**	− 0.03
Restrained eating (NVE) ^a^	− 0.14	− 0.02
BMI	− 0.16	0.13
Waist	− 0.19	0.18
Systolic blood pressure (BP)	− 0.15	0.23
Cholesterol HDL (HDL) ^a^	0.11	− 0.06
Triglycerides (Tri) ^a^	− 0.20	0.14
Glucose	− 0.19	− 0.12

^a^Variable was non-normality distributed, Spearman’s correlation coefficients are displayed. *p < .05, **p < .01

## Discussion

The goal of the current study was to investigate whether mothers and fathers of a young child with ASD display problematic levels of stress, eating behavior and adverse health and evaluate whether parental stress is associated with eating behavior and adverse health. While mothers of a young child with ASD experienced clinical parenting stress (above 90th percentile) five times more often than mothers of neurotypical children, this was three times more often in fathers. Regarding adverse health of mothers in our sample, prevalence rates of obesity (39.1%), abdominal obesity (59.6%) and metabolic syndrome (21.6%) were higher than in same-aged females from the general population. Interestingly, although fathers experienced higher stress as well, there were no significant differences between fathers and the general population regarding adverse health. Parenting stress was related to more emotional eating and external eating in mothers, but not in fathers.

In line with earlier studies, parents of a young child with ASD reported more parenting stress than parents of typically developing children. A meta-analysis by Hayes and Watson (2013), performed in parents of children of all ages, showed a higher experienced parenting stress in parents of a child with ASD than parents of typically developing children or children with another disability. The present study demonstrates that parents of a young child with ASD do not only experience a higher level of parenting stress on average, but also 52% of the mothers and 33% of the fathers of a young child with ASD score above the 90th percentile for parenting stress. While Davis and Carter (2008) reported a similar pattern, the percentages of clinical parenting stress in the study of Davis and Carter are somewhat lower, with 39% of the mothers and 28% of the fathers of a child with ASD scoring above the 90th percentile for parenting stress. Explanatory factors are: the children in the study of Davis and Carter were younger (between 18 and 33 months of age) than the children of the parents in our sample (between 36 and 72 months of age). As each developmental stage of a child comes with different challenges for parents, their experienced stress may differ across the years. Also, all families in the study of Davis and Carter had already received intensive intervention for autism (on average 2 months prior to joining the study), which may have decreased parenting stress in some parents.

To our knowledge, this study is the first study to demonstrate that mothers of a young child with ASD demonstrate higher rates of obesity and metabolic syndrome compared to the general population. This finding is relevant, as having obesity and metabolic syndrome substantially increases the risk for chronic diseases, such as cardiovascular disease and some types of cancer (Blüher, 2019; Galassi et al., 2006). Fairthorne and colleagues (2014) showed higher mortality hazard ratios in mothers of children with ASD and higher likelihood to die from cancer compared to mothers of typically developing children and hypothesized that this association may be mediated by increased stress levels in these mothers or maternal conditions, such as obesity. In line with that hypothesis, our study demonstrated higher rates of obesity and metabolic syndrome in mothers of a child with ASD. Possibly, the high level of stress experienced by the parents in our sample could have contributed to more aberrant eating behavior, leading to weight gain and metabolic health problems, as previous studies suggested a causal relationship between stress and weight gain. (Adam & Epel, 2007; Torres & Nowson, 2007; Wardle et al., 2011). However, based on the data of the current study, we cannot draw conclusions regarding causality of the relationships. Another possibility is that the mothers in our study were already obese before they were pregnant. A meta-analysis by Lei and colleagues (2019) demonstrated an association between maternal obesity before pregnancy and an increased risk for ASD in the child. However, the prevalence rates of maternal obesity before pregnancy in mothers of a child with ASD were lower in the meta-analysis of Lei and colleagues (ranging from 9 to 32%) than the obesity rates in the current study. It is likely that the high prevalence rates in the mothers in our study is due to a combination of the two: prevalence rates of obesity might have been already increased before mothers were pregnant, and mothers may have gained weight after their child was born due to stress.

The current study was the first study to investigate adverse health in fathers of a child with ASD using physiological measures. Although we did not find a higher risk of obesity, hypertension and metabolic syndrome in fathers of a young child with ASD than same-aged males from the general population, this does not automatically mean that fathers do not have a higher risk for adverse health. There are some other possible explanations for this finding. First, fathers of children with ASD spend approximately 26% less time in childcare than mothers (Hartley et al., 2014). Therefore, fathers could be less exposed to parenting stress than mothers. Second, fathers may have different coping mechanisms than mothers with different effects on their health. While some mothers may use (over)eating as a coping mechanism for stress, fathers may have other ways to cope with parenting stress, such as spending time in paid employment. For example, a Swedish study found a positive association between paid-employment and psychological well-being in fathers of children with an intellectual disability (Olsson & Hwang, 2006). Lastly, the observed differences between fathers of a young child with ASD and the general population may increase over time, as stress can have a long-term impact on the health via alterations in the immune system and microbiome imbalance (reviewed by Dijkstra – de Neijs et al., 2020). Therefore, it would be relevant to follow-up the fathers in our sample in a few years to investigate whether the difference between fathers of a child ASD with and the general population has increased. In addition, our finding that one third of the fathers in our sample experienced a clinical level of stress, underlines the need to draw more attention to the well-being of fathers of children with ASD in research and clinical practice.

We found a positive association between parenting stress and emotional and external eating in mothers of a young child with ASD, which is in line with earlier studies which indicated that ongoing stress can lead to chronically stimulated eating behavior (Groesz et al., 2012; Sominsky & Spencer, 2014). Previously, Radin and colleagues (2019) demonstrated a higher level of stress-related eating in parents of a child with ASD than in parents of typically developing children. Radin and colleagues (2019) theorized that chronic stress related to caring for a child with ASD may promote eating behavior on the short term, which may contribute to weight gain and worsening of metabolic components on the long-term. The current study is in line with Radin and colleagues (2019) by demonstrating a positive relationship between parenting stress and emotional eating (eating in response of emotions) and external eating (eating in response of external stimuli) in mothers of a young child with ASD. In addition, the higher rates of obesity and metabolic syndrome in mothers of a child with ASD display the same pattern as theorized by Radin and colleagues. However, as we cannot make causal conclusions based on our data, we encourage future studies to further investigate these trajectories longitudinally.

The current study had some limitations. First, the design of this study was cross-sectional and therefore, we were not able to draw causal conclusions about the development of stress and adverse health in parents of a child with ASD. Another limitation of the study was that we did not have a control group. Therefore, we used information provided by existing norm-groups or databases, which had a few drawbacks. For example, as the norm group on the OBVL consisted of mothers only, we had to compare parenting stress of fathers of children with ASD with mothers of neurotypical children. As there was no specific information available regarding family composition in the Lifelines Cohort Study, we can only draw conclusions about the difference between mothers in our sample and woman from the general population (with and without children). Previous studies show that becoming a mother is associated with a larger increase in weight than remaining childless (Corder et al., 2020). However, an American National Health study did not find a difference between mothers with a young child (aged 0–5 years) versus women without children (Neshteruk et al., 2022). Given the large differences we found between mothers in our sample and the comparison group and the results from previous studies, we think it is very likely that the differences we found can (partly) be attributed to being a parent of a child with ASD specifically. Strengths of the current study were, firstly, the integrated approach, in which concurrently mental- and physical measures were examined. Such an approach allows us to not only measure experiences of parents, but also to measure adverse health in these parents by physiological measurement. A second strength of this study is that it also included fathers, which helped us to increase our knowledge of risk for stress in families of children with ASD. Lastly, all parents participating in this study had a child between 3 and 7 years old who recently received a diagnosis, which enables us to investigate the early dynamics of stress, eating behavior and adverse health in parents of a child with ASD.

In conclusion, the current study evaluated the level of stress, eating behavior and adverse health in parents of a young child with ASD at the age of 3 until 6 years. Considering more than half of the mothers and a one third of the fathers of a young child with ASD experience a clinical level of parenting stress, parents of young children with ASD are an at-risk group for stress. In mothers, we found higher rates of obesity and metabolic syndrome compared to the general population and a correlation between parenting stress and displayed eating behavior. Although we cannot make any causal conclusions based on cross-sectional data, these high parenting stress levels in both parents in combination with the high rates of obesity and metabolic syndrome in mothers are alarming. While most mothers in our sample are between 25 and 40 years old, they already display health problems which put them at higher risk for serious health conditions later in life. It could be theorized that stress, disinhibited eating behavior and adverse health in mothers are interrelated to each other: mothers may use eating as a strategy to cope with their stress, leading to weight gain and adverse health later in life. However, this should be studied longitudinally to test for causal relationships. Even though we did not find adverse health problems in fathers in the current study, this does not mean fathers are not at-risk for developing health problems, as there may be health risks that could emerge later in life. We did find a clinical level of parenting stress in one third of the fathers, which emphasizes the necessity to draw more attention to fathers in research and clinical practice, which is currently often predominantly directed at mothers. In addition, given the high level of stress in both parents, it is important to specifically target parenting stress when treating ASD, for example using group therapy for parents directed at reducing stress and preventing health problems by promoting a healthy lifestyle.
